# The mTOR signal regulates myeloid-derived suppressor cells differentiation and immunosuppressive function in acute kidney injury

**DOI:** 10.1038/cddis.2017.86

**Published:** 2017-03-23

**Authors:** Chao Zhang, Shuo Wang, Jiawei Li, Weitao Zhang, Long Zheng, Cheng Yang, Tongyu Zhu, Ruiming Rong

**Affiliations:** 1 Department of Urology, Zhongshan Hospital, Fudan University, Shanghai, China; 2 Shanghai Key Laboratory of Organ Transplantation, Shanghai, China; 3 Shanghai Public Health Clinical Center, Fudan University, Shanghai, China; 4 Department of Transfusion, Zhongshan Hospital, Fudan University, Shanghai, China

## Abstract

The mammalian target of rapamycin (mTOR) signal controls innate and adaptive immune response in multiple immunoregulatory contexts. Myeloid-derived suppressor cells (MDSCs) are a heterogeneous population of myeloid cells of potent immunosuppressive capacity. In this study, we aimed to investigate the role of MDSCs in the protection of acute kidney injury (AKI) and the regulation of mTOR signal on MDSC's protective role in this context. In mice AKI model, rapamycin administration was associated with improved renal function, restored histological damage and decreased CD4^+^ and CD8^+^ T-cell infiltration in kidney tissue. MDSCs, especially CD11b^+^Ly6G^+^Ly6C^low^ G-MDSCs were recruited to the injured kidney following the interaction of CXCL1, CXCL2 and their receptor CXCR2 after inhibiting mTOR signal with rapamycin treatment. The adoptive transfer of rapamycin-treated MDSCs into the mice with AKI significantly improved the renal function, ameliorated histologic damages and limited the infiltration of T cells in kidney tissue. In addition, the expression of pro-inflammatory cytokines IL-1*β* and IFN-*γ* mRNA was downregulated while the expression of TGF-*β*1 and Foxp3 mRNA was upregulated in kidney tissue after transferring rapamycin-treated MDSCs. Adoptive transfer of rapamycin-treated MDSCs also downregulated the serum levels of IL-1*β*, IL-6 and IFN-*γ* and upregulated the serum levels of TGF-*β*1 compared with the IR group and PBS-treated MDSC group. In *in vitro* study, inhibiting mTOR signal regulated the induction of MDSC towards the CD11b^+^Ly6G^+^Ly6C^low^ G-MDSC subset. The ability to suppress T-cell proliferation of both bone marrow–derived CD11b^+^Ly6G^+^Ly6C^low^ G-MDSCs and CD11b^+^Ly6G^-^Ly6C^high^ M-MDSCs was enhanced by mTOR signal inhibition via upregulating the expression of Arginase-1 and iNOS. Accordingly, both G-MDSCs and M-MDSCs presented downregulated *runx1* gene expression after rapamycin treatment. Taken together, our results demonstrated that MDSCs ameliorated AKI and the protective effect was enhanced by mTOR signal inhibition via promoting MDSCs recruitment, regulating the induction of MDSCs and strengthening their immunosuppressive activity.

Acute kidney injury (AKI) is a common and severe clinical problem with a high incidence of morbidity and mortality.^1^ It is reported that 13.3 million individuals are afflicted with AKI around the globe each year, of whom 1.7 million die of renal failure or multiple organ dysfunction syndrome (MODS) secondary to AKI.^2, 3^ Recent researches into the pathophysiologic mechanism of AKI pointed out that the immune system, both the innate and adaptive immunity, was among the key factors in the pathogenesis of AKI. Various immune cells, including dendritic cells, natural killer T cells, T and B lymphocytes, neutrophils and macrophages are involved.^4, 5, 6^ Of note, T lymphocytes are well established to participate in the early phase of injury.^7^ Studies showed that athymic mice and CD4^–/–^ mice were protected from AKI while adoptive transfer of T cells restored injury.^8^ Another study in which T-cell CD28-B7 costimulatory pathway was blocked by anti-B7-1 antibody provided further evidence that T cells were early mediators of injury.^9^ Therapies that prevent T-cell infiltration may serve as potential interventions to improve the outcomes.

Myeloid-derived suppressor cells (MDSCs) are a heterogeneous population of cells generally composed of progenitors and precursors of dendritic cells, macrophages and granulocytes at various stages of differentiation.^10, 11^ This cell population could exhibit potent immunosuppressive capacity by the upregulation of immune suppressive factors such as Arginase-1 (Arg-1) and inducible nitric oxide synthase (iNOS) both *in vivo* and *in vitro.*^12, 13, 14^ In mice, MDSCs are uniformly identified by co-expression of surface markers CD11b and Gr-1, but with two subtypes based on their distinct expression of Ly-6C and Ly-6G.^15, 16, 17^ To be specific, granulocytic MDSCs (G-MDSCs) are identified as CD11b^+^Ly6G^+^Ly6C^low^ while monocytic MDSCs (M-MDSCs) are identified as CD11b^+^Ly6G^−^Ly6C^high^.^15^ A growing body of evidences demonstrated that MDSCs had a pivotal role in controlling devastating inflammatory response and/or tissue injury, such as asthma-related airway inflammation,^18^ tumor-related inflammation,^19^ spinal cord injury,^20^ LPS-induced acute lung injury,^21^ autoimmune encephalomyelitis^22^ and immunological hepatic injury,^23^ but few directly explored its role in acute kidney injury.

The mammalian target of rapamycin (mTOR) is an evolutionary conserved and generally expressed serine–threonine kinase that regulates a variety of cellular activities, such as proliferation, differentiation, autophagy, energy maintenance and response to pathogens.^24^ However, the role of mTOR signal in regulating MDSCs has been seldom investigated until a recent finding indicated that inhibition of mTOR signal by rapamycin could promote the recruitment of CD11b^+^Gr1^+^Ly6C^high^ MDSCs to inflammatory sites and mediate protection against murine immunological hepatic injury.^23^ Moreover, the results of another finding suggested that mTOR inhibition prolonged cardiac allograft survival in a mouse model by accumulating and expanding MDSCs in allografts.^25^ Despite the reported advances in this novel field, how mTOR signal affect the differentiation and immunosuppressive function remains largely unclear. Besides, we wonder whether MDSCs could exert a protective effect on acute kidney injury as well. In the present study, we aim to investigate the role of MDSCs in ischemic AKI and how it is regulated by mTOR signaling in this context.

## Results

### Rapamycin protects mice kidney against AKI *in vivo*

**Figure 1:**
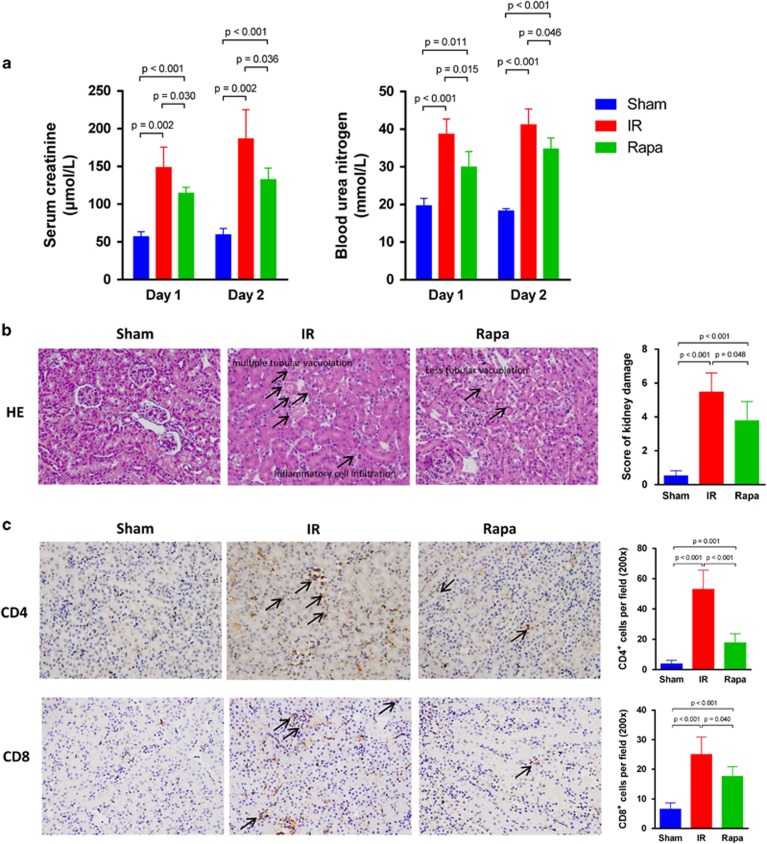
Rapamycin protects mice kidney against AKI *in vivo*. (**a**) The level of serum creatinine (*μ*mol/l) and blood urea nitrogen (mmol/l) on POD 1 and POD 2. (**b**)The histological image of H&E-stained kidney tissue (× 200). Semi-quantitative analysis was performed following histological scoring system. (**c**) Immunohistochemical staining of CD4 and CD8 in kidney tissue (× 200). The black arrow pointed to the positive cells. The average number of positive cells per field was analyzed. (data were shown as mean±S.D.; *n*=5 mice per group per time point; POD, postoperative day)

To investigate the renoprotective effect of rapamycin in mice AKI model, the levels of serum creatinine (Scr) and blood urea nitrogen (BUN) in the blood samples of mice in each group were analyzed by using ELISA kit. Both on postoperative day (POD) 1 and 2, the level of Scr and BUN were significantly increased in the ischemia–reperfusion (IR) group when compared with the sham group. In particular, the level of Scr and BUN were even higher on POD 2 than on POD 1, which was consistent with our previous study that 48 h post operation marked the climax of kidney IR injury.^26^ With rapamycin treatment, however, the level of Scr and BUN declined on both POD 1 and POD 2, showing protective effect of rapamycin against kidney ischemia–reperfusion injury (IRI; Figure 1a). We further examined the role of rapamycin in AKI in a histopathological perspective. Kidney tissues were stained with hematoxylin–eosin and semi-quantitative analysis was performed by using a histological scoring system. The results revealed that the tissue structure of kidney in the rapamycin-treated group was well protected, with mild interstitial edema, fewer protein casts in tubular lumens and tubular epithelial vacuolation or detachment seldom found compared with IR group (the images of hematoxylin and eosin (H&E)-stained kidney tissue were similar in POD 1 and POD 2, so we just displayed one representative image here). The score of kidney injury was decreased accordingly (Figure 1b). We also examined the infiltration of CD4^+^ and CD8^+^ T cells in kidney tissue by immunohistochemical assay. The number of infiltrated CD4^+^ and CD8^+^ T cells in kidney tissue was observed to decrease significantly in the Rapa group compared with IR group (Figure 1c). Because there was little difference of infiltrated numbers of CD4^+^ and CD8^+^ T cells in POD 1 and POD 2, we presented one representative image for each group.

### MDSCs, especially G-MDSCs, recruit to injured kidney following mTOR inhibition

**Figure 2:**
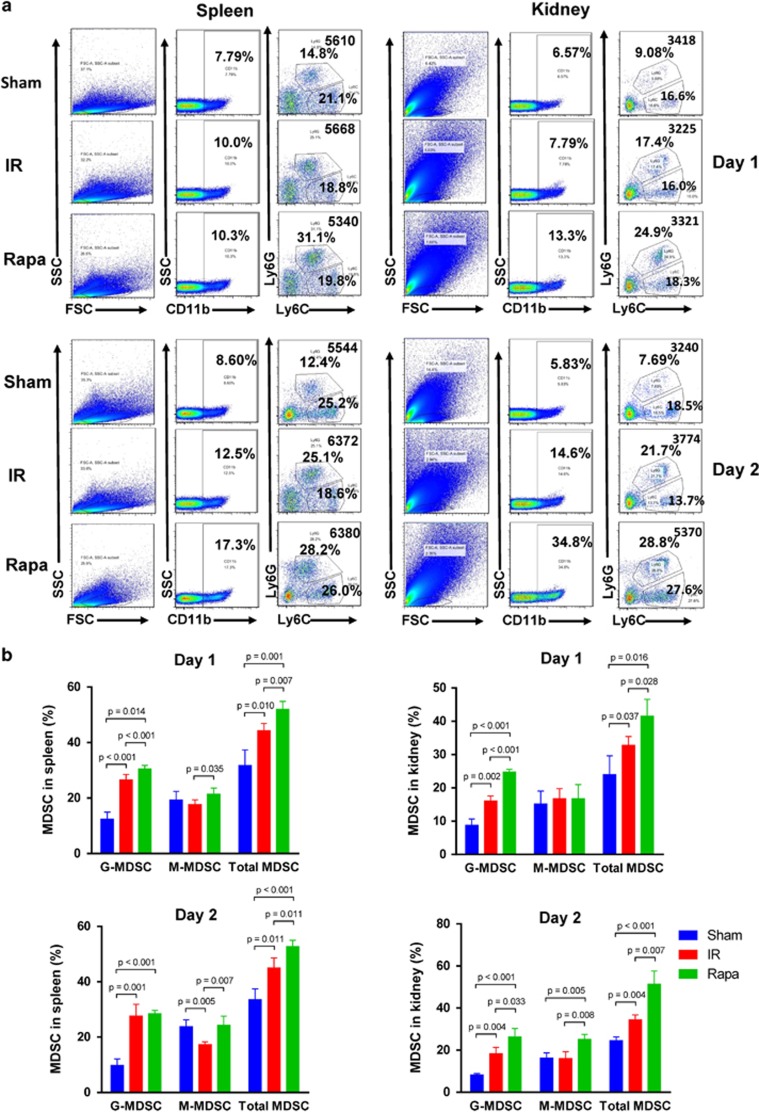
MDSCs recruit to injured kidney following rapamycin treatment. CD11b^+^ cells were first gated from FSC/SSC, and then Ly-6G^+^Ly-6C^low^ and Ly-6G^−^Ly-6C^high^ cell populations were detected within CD11b^+^ cells. (**a**) The frequency of CD11b^+^Ly-6G^+^Ly-6C^low^ G-MDSCs, CD11b^+^Ly-6G^−^Ly-6C^high^ M-MDSCs and total MDSCs in spleen on POD 1 and POD 2 were examined by flow cytometry. (**b**) The frequency of CD11b^+^Ly-6G^+^Ly-6C^low^ G-MDSCs, CD11b^+^Ly-6G^−^Ly-6C^high^ M-MDSCs and total MDSCs in the kidney on POD 1 and POD 2 were examined by flow cytometry. (data were shown as mean±S.D.; *n*=5 mice per group per time point; POD, postoperative day)

To examine how rapamycin treatment altered MDSCs migration *in vivo*, we analyzed the frequency of total MDSCs, CD11b^+^Ly-6G^+^Ly-6C^low^ G-MDSCs and CD11b^+^Ly-6G^−^Ly-6C^high^ M-MDSCs in the spleen and kidney with flow cytometry. Single cell suspension from spleen and kidney were prepared and stained with FITC-labeled CD11b, PerCP-Cy 5.5-labeled Ly-6G and APC-labeled Ly-6C. In the spleen, the frequency of total MDSCs was increased after IR injury on POD 1 and POD 2, and the frequency was even higher with rapamycin treatment. The frequency of G-MDSCs and M-MDSCs was increased in the Rapa group compared with the IR group on POD 1. There also existed a significant increase in the frequency of M-MDSCs in the Rapa group than in the IR group on POD 2, but the frequency of G-MDSCs was not augmented significantly (Figure 2a). In the kidneys, rapamycin treatment augmented the frequency of total MDSCs in comparison with IR group both on POD 1 and on POD 2. The alteration in the frequency of G-MDSCs after rapamycin treatment showed the similar trend. However, a significant increase of the frequency of M-MDSCs was only seen on POD 2 but not on POD 1 (Figure 2b). In summary, the results revealed that mTOR signal inhibition recruited MDSCs, especially G-MDSCS, to IR-inducing kidneys on both POD 1 and POD 2.

### CXCL1, CXCL2 and CXCR2 signal mediates the recruitment of MDSCs in injured kidney

**Figure 3:**
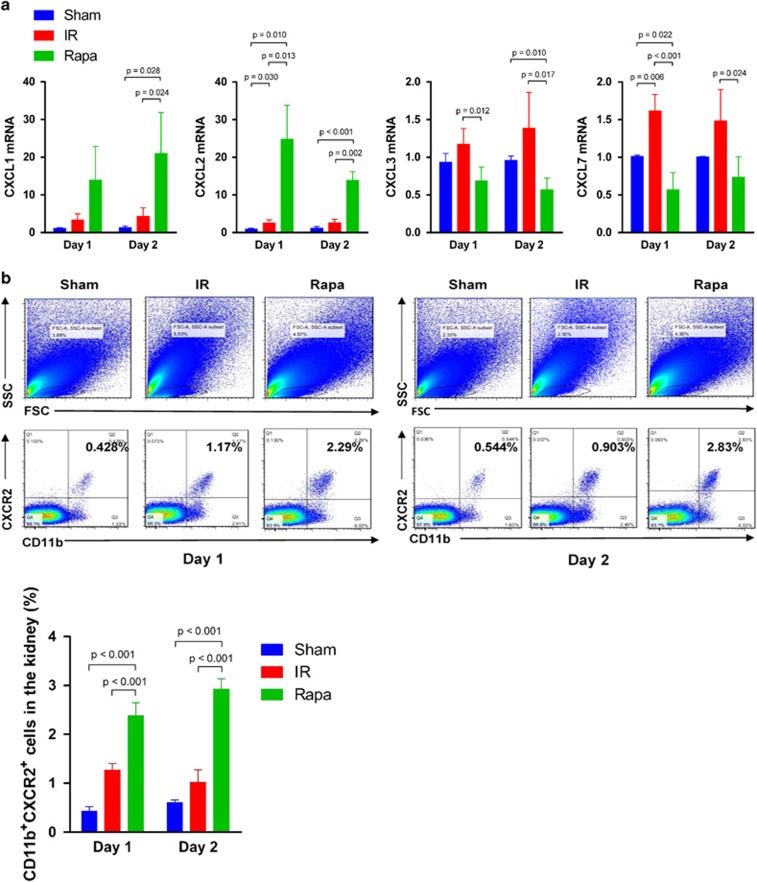
CXCL1, CXCL2 and CXCR2 interaction mediates the recruitment of MDSCs in injured kidney. (**a**) The expression of chemokine CXCL1, CXCL2 mRNA in kidney were upregulated on POD 1 and POD 2, but the expression of CXCL3 and CXCL7 mRNA were not (the expression was normalized to GAPDH and relative to control). (**b**) The percentage of CD11b^+^CXCR2^+^ cells in the kidney on POD 1 and POD 2 were increased in the rapamycin-treated group compared with sham group and the IR group. Cells in the kidney were gated first in FSC/SSC, and then CD11b^+^CXCR2^+^ cells were detected by flow cytometry. (data were shown as mean±S.D.; *n*=5 mice per group per time point; POD, postoperative day)

Then we further examined which factors contributed to the recruitment of MDSCs in IR-induced kidney. The expression of chemokines CXCL1, CXCL2, CXCL3 and CXCL7, which showed potentials to drive MDSCs or myeloid cells migration,^27, 28, 29^ was examined by reverse transcription-quantitative polymerase chain reaction(RT-qPCR) in kidney tissues on POD 1 and 2. The results showed that on both POD 1 and POD 2, the mRNA expression of CXCL1 and CXCL2 were significantly increased in rapamycin-treated group compared with IR group while the mRNA level of CXCL3 and CXCL7 were decreased following rapamycin treatment, indicating that CXCL1 and CXCL2 had an important role in mediating MDSC recruitment (Figure 3a). Accordingly, the expression of CXCR2, the receptor of the chemokines CXCL1 and CXCL2, was also upregulated in the kidney in the Rapa group in comparison with the IR group on both POD 1 and 2 (Figure 3b). Thus, the results indicated that it was the signal of CXCL1, CXCL2 and their receptor CXCR2 that mediated MDSCs' recruiting to the injured kidney to exert protective effects.

### Adoptive transfer of MDSCs protects kidney against AKI and mTOR signal inhibition enhances MDSCs' protective effects

**Figure 4:**
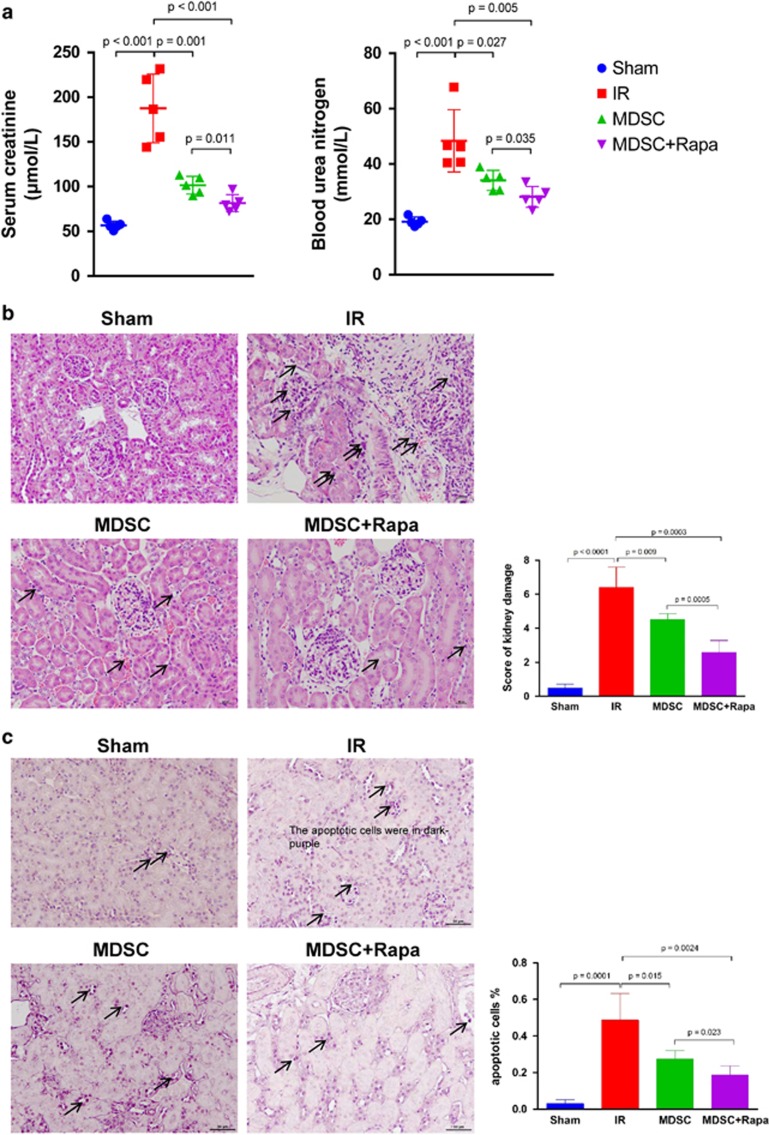

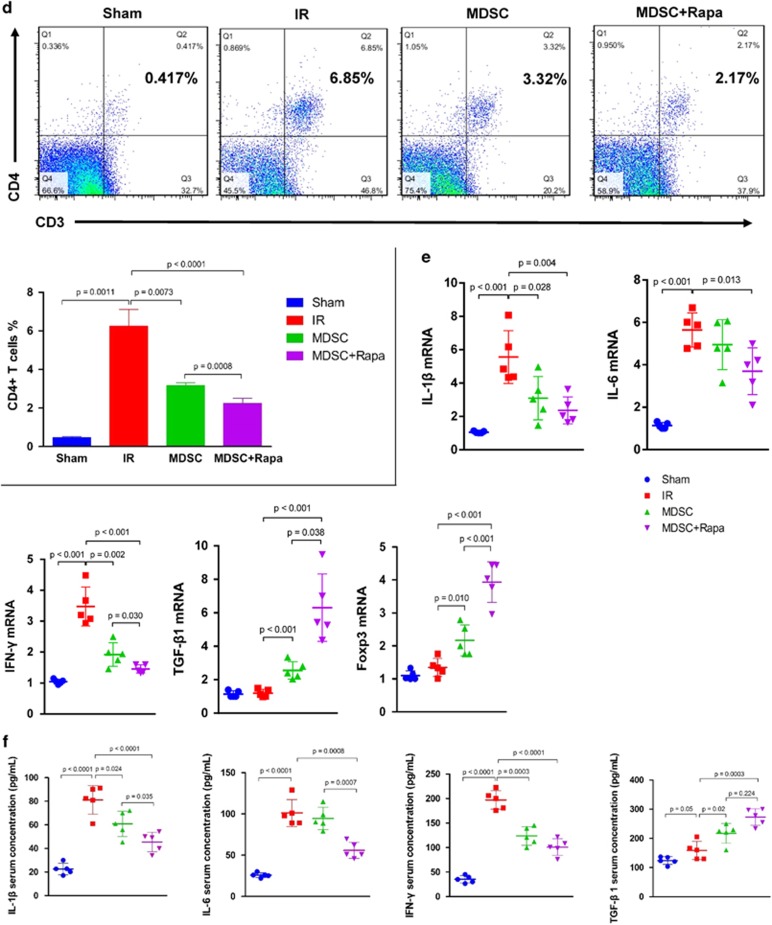
Adoptive transfer of MDSCs protects kidney against AKI and mTOR signal inhibition enhances MDSCs' protective effects. (**a**) The level of serum creatinine (*μ*mol/l) and blood urea nitrogen (mmol/l) were decreased after adoptive transfer of MDSCs and further decreased after adoptive transfer of rapamycin-treated MDSCs. (**b**) Renal histologic damage was assessed in H&E-stained sections. Mild-to-moderate tubular vacuolation and hemorrhage were observed in the kidneys in the MDSC group compared with the IR group, and the histologic damage of kidneys in Rapa+MDSC group was even milder. (**c**) The percentage of apoptotic cells (shown as dark purple in color) in kidney tissues *in situ* was detected and quantified by using TUNEL assay. (**d**) Flow cytometry analysis showed the percentage of infiltrated CD4+ T cells in kidney tissues after adoptive transfer of non-rapamycin-treated MDSCs and rapamycin-treated MDSCs. (**e**) The expression of IL-1*β*, IL-6, IFN-*γ*, TGF-*β*1 and Foxp3 mRNA in the kidney was examined by RT-qPCR. (**f**) The serum level of cytokine IL-1*β*, IL-6, IFN-*γ* and TGF-*β*1 was measured by using Luminex and ELISA kit. (data were shown as mean±S.D.; *n*=5 mice per group; IHC, immunohistochemistry; RT-qPCR, reverse transcription-quantitative polymerase chain reaction; TUNEL, TdT-mediated dUTP Nick-End Labeling)

To investigate the role of MDSCs in the protection of AKI and to verify whether the mTOR signal is involved in MDSCs' protective effects, bone marrow (BM)-derived MDSCs with or without rapamycin treatment were adoptively transferred into the mice via tail vein. We first analyzed the renal function of mice in four groups on POD 1. Compared with the IR group, the level of serum creatinine (Scr) and blood urea nitrogen (BUN) was decreased after adoptive transfer of MDSCs. Furthermore, mice injected with rapamycin-treated MDSCs presented even lower level of Scr and BUN, indicating that inhibition of mTOR signal enhanced MDSCs' protection effect to AKI (Figure 4a). The renal histological assessment in each group was performed by H&E staining. Semi-quantitative analysis revealed that the tissue structure was protected after adoptive transfer of MDSCs, and even better protected after adoptive transfer of rapamycin-treated MDSCs (Figure 4b). The rate of apoptotic cells was examined by using *in situ* end-labeling (ISEL) assay. In the MDSC-transferred group, the rate of ISEL+ apoptotic cells were dramatically reduced in the kidney post IR injury. In Rapa-MDSC-transferred group, however, the rate of apoptotic cells in kidney was further decreased (Figure 4c). We then examined the level of CD4^+^ T-cell infiltration in kidneys transferred by the MDSCs with or without rapamycin treatment. Single cell suspension from kidney tissues were prepared and stained with anti-CD3 and anti-CD4 antibody. The percentage of infiltrated CD4^+^ T cells in kidney was detected by flow cytometry. The results showed that after IR injury, the number of infiltrated CD4^+^ T cells was significantly increased. However, adoptive transfer of MDSCs ameliorated T-cell infiltration. After transfer of rapamycin-treated MDSCs, the level of CD4^+^ T-cell infiltration in kidney was further decreased (Figure 4d). The mRNA expression of IL-1*β*, IL-6, IFN-*γ*, TGF-*β*1 and Foxp3 in kidney were examined by RT-qPCR. The results showed that IR-induced kidneys expressed augmented level of pro-inflammatory cytokines IL-1*β* and IFN-*γ*. After adoptive transfer of MDSCs, however, the expression of these pro-inflammatory cytokines were downregulated while the expression of anti-inflammatory cytokine TGF-*β*1 was upregulated. Adoptive transfer of rapamycin-treated MDSCs further lowered IL-1*β* and IFN-*γ* mRNA levels and augmented TGF-*β*1 mRNA level in the kidney tissue. IL-6 expression was not significantly reduced by transfer of MDSCs or Rapa-MDSCs. The level of Foxp3 mRNA was increased following adoptive transfer of MDSCs and even higher in Rapa-MDSC-treated group (Figure 4e). The serum level of cytokine IL-1*β*, IL-6, IFN-*γ* and TGF-*β*1 was measured by using Luminex and ELISA kit. The results showed the downregulation of IL-1*β*, IL-6 and IFN-*γ* serum concentration and the upregulation of TGF-*β*1 serum concentration in rapamycin-treated MDSC-transferred group in comparison with PBS-MDSC-transferred group (Figure 4f).

### The mTOR signal regulates MDSCs subsets differentiation

**Figure 5:**
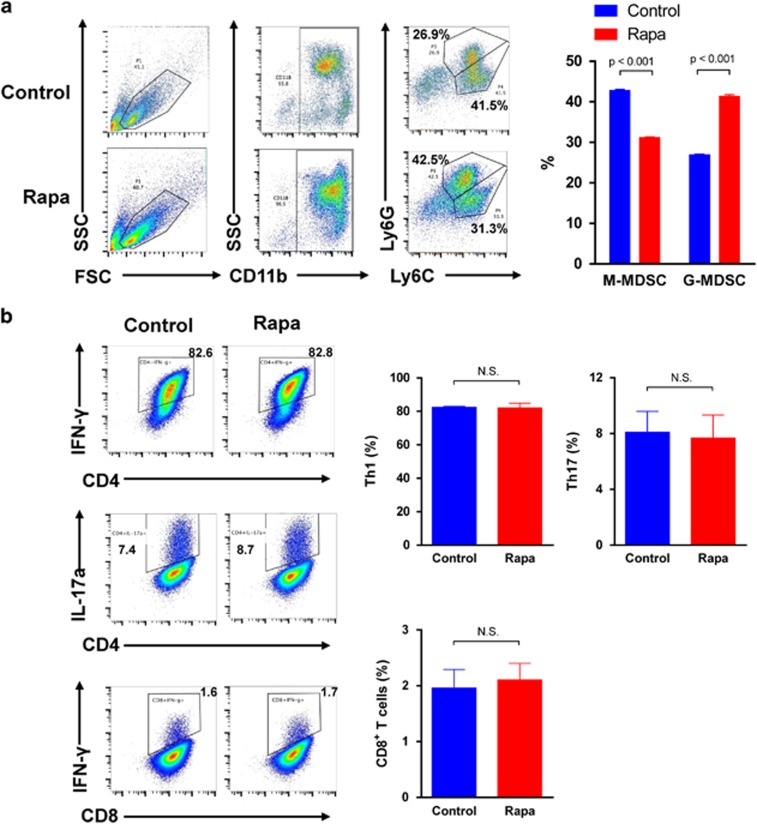
mTOR signal regulates the induction of MDSCs from bone marrow cells. (**a**) CD11b^+^ cells were gated first, and Ly-6G^+^Ly-6C^low^ and Ly-6G^−^Ly-6C^high^ cell populations were detected within CD11b^+^ cells. In comparison with control, rapamycin treatment directed MDSC induction towards Ly-6G^+^Ly-6C^low^ G-MDSC subtype. (**b**) After 100 nM rapamycin or equal volume of DMSO treatment for 4 h, the frequencies of CD4^+^IFN-*γ*^+^ Th1 cells, CD4^+^IL-17a^+^ Th17 cells and CD8^+^IFN-*γ*^+^ T cells were examined by using flow cytometry, respectively. (data were shown as mean±S.D.; *n*=5 duplicate tests)

To investigate whether MDSCs differentiation is regulated by mTOR signal, we treated BM cells with 100 nM rapamycin combined with GM-CSF+IL-6. In the control group, BM cells were treated with equal volume of DMSO. Then the two subtypes of MDSCs were tested by flow cytometry. Compared with the control group, the percentage of CD11b^+^Ly-6G^+^Ly-6C^low^ G-MDSCs was upregulated while the percentage of CD11b^+^Ly-6G^−^Ly-6C^high^ M-MDSCs was downregulated with mTOR signal inhibition, which indicated that mTOR inhibition redirected the differentiation towards G-MDSCs (Figure 5a). Then we examined whether rapamycin had direct effect on pro-inflammatory T-cell subsets. Naive T cells were isolated from mononuclear splenocytes and then activated with anti-CD3 and anti-CD28 monoclonal antibodies. The activated T cells were treated with 100 nM rapamycin or equal volume of DMSO for 4 h. The frequencies of CD4^+^IFN-*γ*^+^ Th1 cells, CD4^+^IL-17a^+^ Th17 cells and CD8^+^ IFN-*γ*^+^ T cells in the control and rapamycin-treated group were examined by using flow cytometry, respectively. We found that there were no significant differences between the rapamycin-treated group and the control group with regard to the frequencies of CD4^+^ IFN-*γ*^+^ Th1 cells, CD4^+^IL-17a^+^ Th17 cells and CD8^+^IFN-*γ*^+^ T cells (Figure 5b). This examination indicated that rapamycin has little direct effect on T-cell subsets *in vitro* and the suppressive role of rapamycin on T cells was mediated by MDSCs.

### Inhibition of mTOR promotes immunosuppressive activity of MDSCs

**Figure 6:**
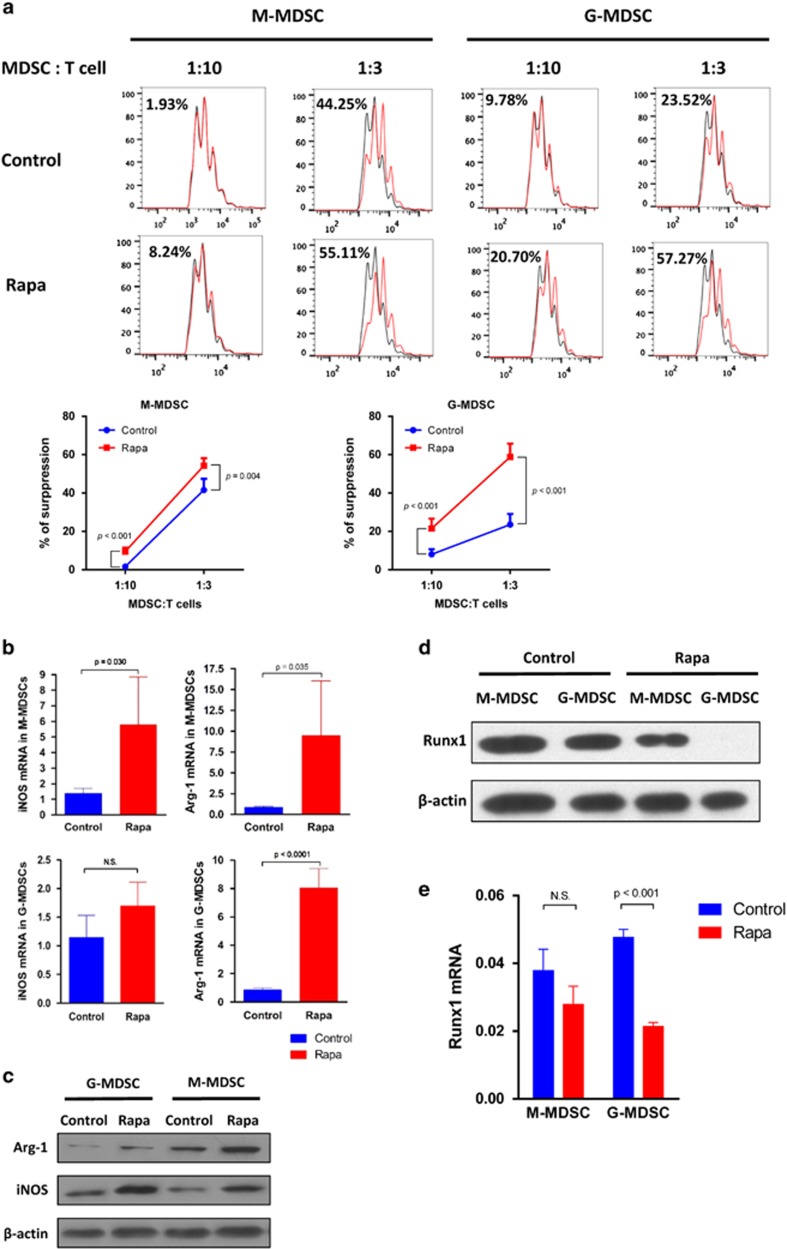
mTOR inhibition with rapamycin promotes immunosuppressive activity of MDSCs *in vitro*. (**a**) CD11b^+^Ly-6G^+^Ly-6C^low^ G-MDSCs and CD11b^+^Ly-6G^−^Ly-6C^high^ M-MDSCs with DMSO or rapamycin treatment were co-cultured with CFSE-stained CD4^+^ T cells at the ratio of 1:10 and 1:3 for 5 days, respectively, then the proliferation of T cells was assayed by flow cytometry. The decreased percentage of CFSE MFI after DMSO or rapamycin treatment indicated the ability of suppression of T-cell proliferation. Rapamycin treatment promoted immunosuppressive function of both M-MDSCs and G-MDSCs compared with control. (**b**) Arginase-1 and iNOS mRNA expression in CD11b^+^Ly-6G^−^Ly-6C^high^ M-MDSCs and CD11b^+^Ly-6G^+^Ly-6C^low^ G-MDSCs were examined by RT-PCR. The expression of Arginase-1 and iNOS mRNA were upregulated in M-MDSCs, whereas in G-MDSCs only Arginase-1 mRNA was upregulated after rapamycin treatment. (**c**) Arginase-1 and iNOS protein levels in CD11b^+^Ly-6G^+^Ly-6C^low^ G-MDSCs and CD11b^+^Ly-6G^−^Ly-6C^high^ M-MDSCs were examined by western blot. For both G-MDSCs and M-MDSCs, the protein levels of Arginase-1 and iNOS were increased after rapamycin treatment when compared with the control group. (**d**)The protein levels of Runx1 in G-MDSCs and M-MDSCs with or without rapamycin treatment were examined by western blot. The levels of Runx1 protein in both G-MDSCs and M-MDSCs were significantly reduced after rapamycin treatment in comparison with the control groups. There was almost no Runx1 expression in G-MDSCs after rapamycin treatment. (**e**) The levels of runx1 mRNA in G-MDSCs and M-MDSCs were examined by RT-PCR. Both G-MDSCs and M-MDSCs presented downregulated expression of runx1 mRNA after rapamycin treatment, however, the decrease of runx1 mRNA in M-MDSCs was not significant. (data were shown as mean±S.D.; *n*=5 duplicate tests; Arg-1, Arginase-1; CFSE, carboxyfluorescein succinimidylester; DMSO, dimethyl sulfoxide; MFI, mean fluorescence intensity)

The key characteristic of MDSCs is immunosuppressive function. Therefore, we further explored whether mTOR signal regulates immunosuppressive activity of MDSCs. After induction by GM-CSF+IL-6 for 4 days, CD11b^+^Ly-6G^+^Ly-6C^low^ G-MDSCs and CD11b^+^Ly-6G^−^Ly-6C^high^ M-MDSCs were sorted from BM cells first and then treated with 100 nM rapamycin or DMSO for 4 h, respectively. After DMSO or rapamycin treatment, M-MDSCs and G-MDSCs were co-cultured with CFSE-labeled activated T cells at the ratio of 1:10 and 1:3 for 5 days, respectively, then the T-cell proliferation assay was performed by flow cytometry. After rapamycin treatment, both M-MDSCs and G-MDSCs displayed a significantly enhanced immunosuppressive effect on CD4^+^ T-cell proliferation than that seen in control group *in vitro*. In addition, we found that the suppressive activity of G-MDSCs were promoted to a greater extent than that of M-MDSCs at the ratio of 1:3 after rapamycin treatment (Figure 6a). To investigate the mechanism mediating enhanced immunosuppressive function by rapamycin, the expression of iNOS and Arginase-1 (Arg-1) mRNA in G-MDSCs and M-MDSCs were examined by PT-PCR. It showed that M-MDSCs expressed a significantly increased level of iNOS and Arg-1, whereas G-MDSCs just expressed increased levels of Arg-1 (Figure 6b). Accordingly, the protein levels of Arg-1 and iNOS in G-MDSCs and M-MDSCs were examined by western blot. The result showed that compared with the control group, rapamycin significantly augmented the expression of Arg-1 and iNOS protein in both G-MDSCs and M-MDSCs (Figure 6c). In sum, rapamycin enhanced immunosuppressive activity of both G-MDSCs and M-MDSCs *in vitro* by upregulating the expression of Arg-1 and iNOS. The gene of runt-related transcription factor 1 (runx1) was demonstrated to involve in the differentiation and suppressive activity of MDSCs. The knockdown of Runx1 in MDSCs results in enhanced capacity of inhibiting T-cell proliferation by promoting the expression of Arg-1 and iNOS.^30^ Therefore, we examined the level of Runx1 protein in BM-derived G-MDSCs and M-MDSCs with or without rapamycin treatment. We found that the levels of Runx1 protein in both G-MDSCs and M-MDSCs were significantly reduced after the rapamycin treatment (there was almost no Runx1 expression in G-MDSCs after rapamycin treatment) in comparison with the control groups (Figure 6d). Accordingly, the mRNA levels of gene runx1in G-MDSCs and M-MDSCs were also examined by RT-PCR. The results showed that both G-MDSCs and M-MDSCs presented downregulated expression of runx1 mRNA after rapamycin treatment, though the decrease of runx1 mRNA in M-MDSCs was not significant (Figure 6e).

## Discussion

As MDSCs have shown great potential in regulating inflammation and ameliorating tissue injury caused by inflammatory responses,^18, 19, 20, 21, 22, 23^ it is rational to think that strategies to promote the accumulation and function of MDSCs may provide new therapeutic options for protecting injured tissues and organs. Here, we showed that inhibition of mTOR signal enhanced MDSCs' protective effect against acute kidney injury in mice model.

It has been demonstrated that severe AKI could be protected by targeting the mTOR signaling pathway, the mechanism of which involved inhibiting the apoptosis of tubular epithelial cells (TECs), inducing autophagy, limiting the infiltration of pro-inflammatory cytokines and enriching CD4^+^CD25^+^Foxp3^+^ regulatory T cells in injured tissue.^31, 32, 33, 34^ However, how mTOR signal regulates MDSCs in the context of AKI was not fully understood so far. In the present study, we first confirmed that rapamycin indeed protected against acute ischemic injury within 2 days after operation, which was associated with inhibited apoptosis of TECs and decreased T-cell infiltration. The reason why we chose the time point as POD 1 and POD 2 was that our previous study revealed that rapamycin attenuated renal IR injury at the early stage but aggravated it at the late stage as the result of impaired recovery from acute renal failure. Then our study showed that rapamycin treatment increased the frequencies of total MDSCs and the two subtypes, CD11b^+^Ly6G^+^Ly6C^low^ G-MDSCs and CD11b^+^Ly6G^-^Ly6C^high^ M-MDSCs, in spleen and kidney tissue. Particularly, we found that it was G-MDSCs, not M-MDSCs, which significantly accumulated in IR-induced kidney on both POD 1 and POD 2. This is of vital significance as G-MDSCs are able to induce unresponsiveness of T cells to specific antigens: peroxynitrite produced by G-MDSCs leads the nitration of T-cell receptors (TCRs), thereby blocking TCR/MHC-peptide specific recognition.^35, 36^ Thus, our study suggested that the recruitment of rapamycin-induced MDSCs, especially G-MDSCs was at least partially responsible for the amelioration of acute kidney injury. The chemoattractant factors released by injured cells may recruit multiple immune cells in the course of inflammatory response. In this study, we showed rapamycin treatment upregulated the expression of the chemokines CXCL1 and CXCL2 that mediate MDSCs recruitment during kidney injury. Accordingly, a significantly higher level of CXCR2 (the receptor of CXCL1 and CXCL2) expression was observed in the rapamycin-treated kidney tissues. Taken together, the results suggested that rapamycin treatment promoted the interaction of CXCL1, CXCL2 and their receptor CXCR2, which contributed to MDSCs recruitment to the kidney.

In addition to promoting MDSCs accumulation in the site of injury, we still wonder how mTOR signal regulates MDSCs directly *in vitro* and whether or not the regulation of mTOR signal has influence on MDSCs protection in AKI. In this study, BM-induced MDSCs were first treated with rapamycin *in vitro* and then adoptive transferred into mice model of renal IRI. Our data showed that administration with rapamycin-treated MDSCs were associated with better renal function, improved histologic damage and decreased T-cell infiltration in kidney compared with transfer of MDSCs without rapamycin treatment, which revealed that inhibition of mTOR signal in MDSCs heightened their protective role in AKI. Meanwhile, transfer of Rapa-MDSCs downregulated the expression of pro-inflammatory cytokines IL-1*β* and IFN-*γ* while the expression of IL-6 was not affected in a significant way. TGF-*β*1 was one of the key molecules secreted by MDSCs to exert suppressive activity,^37, 38^ the expression of which was further elevated after mTOR signal inhibition in this study. In addition, the increased expression of foxp3 mRNA in kidney tissue indicated more Tregs present in injured kidneys by MDSCs and rapamycin-treated MDSCs transfer. However, whether MDSCs could induce Tregs in this condition still needs further research.

Then we investigated the possible mechanism mediating enhanced protective role of MDSCs by rapamycin. It has been reported that CD11b^+^Ly6G^+^Ly6C^low^ G-MDSC is the main MDSC subset to be expanded in tumor animal models.^14^ Similarly, our data showed that the induction of MDSCs was redirected towards the CD11b^+^Ly6G^+^Ly6C^low^ G-MDSC subset following mTOR signal inhibition, indicating that G-MDSCs had a more important role in improving kidney injury. Moreover, both G-MDSCs and M-MDSCs showed improved activity of suppressing T-cell proliferation *in vitro* following mTOR signal inhibition, which accounted for the significantly decreased T-cell infiltration in injured kidney after adoptive transfer of Rapa-MDSCs. Arginase-1 and iNOS serve as key effectors in mediating the suppressive function of MDSCs.^36, 39, 40^ Both Arginase-1 and iNOS use l-arginine as a substrate to produce urea and l-ornithine or NO, respectively. The upregulation of Arginase-1 in MDSCs could lead to the depletion of l-arginine in the microenvironment, which induces the loss of the CD3*ζ* chain and prevents the upregulation of cell cycle regulator in T cells resulting in an inhibition of T-cell proliferation. The upregulation of iNOS activity could lead to the increased NO production, which was shown to suppress T-cell proliferation and function. NO is able to inhibit the downstream pathway of IL-2 receptor by blocking the phosphorylation of Jak3/Stat5 or directly induce the apoptosis of T cells.^36^ In our study, inhibition of the mTOR signal enhanced immunosuppressive activity of MDSCs via upregulating Arginase-1 and iNOS mRNA and protein levels. Runt-related transcription factor 1 (Runx1) is an essential transcription factor that masters the formation and development of multiple hematopoietic stem cells and induces the differentiation of myeloid, lymphoid and megakaryocytic lineages.^41^ Recently, Tian *et al.*^30^ demonstrated that Runx1 had an important role in promoting MDSC differentiation into more mature myeloid cells. Moreover, levels of Arginase-1 and iNOS secreted by M-MDSCs and G-MDSCs were markedly upregulated after Runx1 was knocked down. Knocking down Runx1 expression in both M-MDSCs and G-MDSCs significantly enhanced the capacity of MDSCs to suppress CD4^+^ and CD8^+^ T-cell proliferation.^30^ In our study, both BM-derived G-MDSCs and M-MDSCs displayed a significantly decreased expression of Runx1 mRNA and protein after treatment with rapamycin in comparison with control group, which was consistent with the elevated expression of iNOS and Arginase-1 expression in both M-MDSCs and G-MDSCs. Our study indicated that the gene *runx1* was involved in the enhanced immunosuppressive function of MDSCs induced by rapamycin treatment.

**Figure 7:**
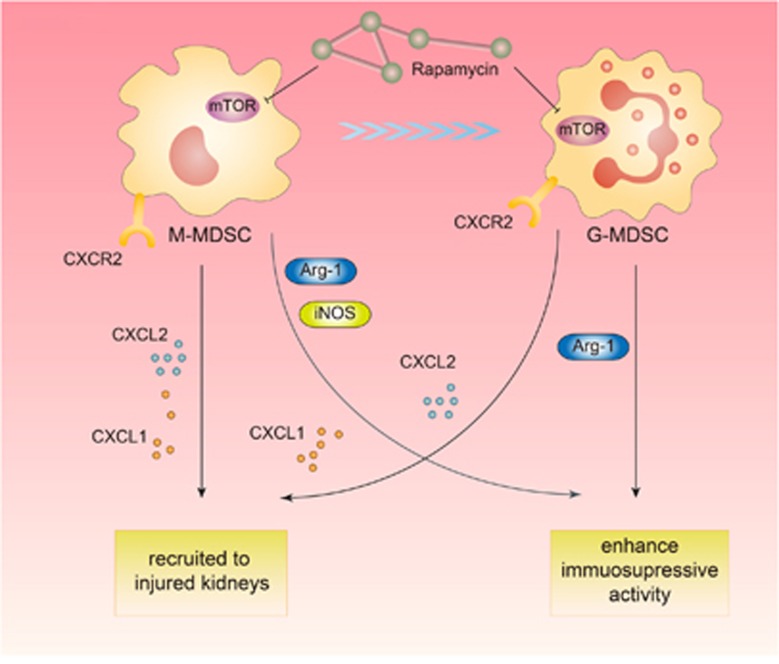
The schematic figure explicating the regulation of mTOR signal on myeloid-derived suppressor cells in acute kidney injury. Inhibition of mTOR signal with rapamycin enables to enhance the protective role of MDSCs via promoting MDSCs recruitment to injured kidney, regulating the induction of MDSCs from bone marrow cells and strengthening its immunosuppressive activity

In conclusion, the results of the present study for the first time demonstrate that BM-derived MDSCs have a protective effect on acute kidney injury in mice model. Inhibition of mTOR signal with rapamycin enables to enhance the protective role of MDSCs via promoting MDSCs recruitment to injured kidneys, regulating the induction of MDSCs from BM cells and strengthening its immunosuppressive activity *in vitro* (Figure 7). Further research is still needed to determine the activation of specific downstream pathways as well as changes in intracellular biological behaviors following mTOR inhibition that mediate the altered characteristics of MDSCs. The more profound understanding of the precise control of MDSCs by mTOR signal will facilitate the clinical application of MDSCs in AKI treatment in the future.

## Materials and methods

### Animals, experiment grouping and renal ischemia–reperfusion injury model

Six-week-old male C57BL/6 mice (weighing 20–25 g), were purchased from Shanghai Slac Lab Animal, Co. Ltd (Shanghai, China), and bred in an experimental animal room of SPF grade in Experimental Animal Center of Zhongshan Hospital, Fudan University. All the animal procedures were performed according to the guidelines of the Care and Use of the Laboratory Animal Ethical Commission of Fudan University. For *in vivo* study, 30 male C57BL/6 mice were randomly divided into three groups: (i) sham group in which the abdominal cavity of mice were exposed for 30 min and then closed without any operating on kidney; (ii) IR group in which bilateral kidneys of mice suffered 30 min ischemia by clipping renal pedicles with vascular clamps; (iii) Rapa group in which kidney-injured mice were administered with rapamycin (2.5 ml/kg) by gastric lavage on 48 h and 24 h pre-operation and 1h post-operation. On POD 1 and 2, five mice in each group were executed and the samples were collected. For the study of MDSC adoptive transfer, 20 male C57BL/6 mice were randomly divided into four groups: (i) sham group in which sham-operated mice were injected with 200 *μ*l PBS ; (ii) IR group in which kidney-injured mice were injected with equal volume of PBS; (iii) MDSC group in which kidney-injured mice were injected with 2 × 10^6^ MDSCs suspended in 200 *μ*l PBS; (iv) Rapa+MDSC group in which kidney-injured mice were injected with 2 × 10^6^ rapamycin-treated MDSCs in 200 *μ*l PBS. All the injections were via tail vein of mice at 4 h post-operation. For the renal ischemia–reperfusion model, the mice were anesthetized with pentobarbital at a dosage of 0.1 g/kg body weight intraperitoneally. The core body temperature was maintained at 37 °C by using a heating blanket. The abdominal cavity was exposed via a midline incision, both kidneys were exposed and the renal pedicles were carefully isolated. Bilateral renal occlusion for 30 min was performed by using 12 mm non-traumatic vascular clamps. Occlusion was confirmed by observing blanching of the entire kidney surface. After removing the renal clips, the kidneys were observed for an additional 5 min to ensure color change indicating blood reperfusion. Afterwards, 0.5 ml saline at 37 °C was injected into the abdomen and the incision was sutured in two layers. After the tissue and blood samples were harvested under anesthesia, the mice were killed.

### Obtainment and culture of MDSCs

Tibias and femurs from C57BL/6 mice were removed using sterile techniques and the BM cells were flushed out of the cavity. The red blood cells (RBCs) were lysed with RBC lysis buffer (Tiangen Biotech, Beijing, China). To obtain BM-derived MDSCs, 2.5 × 10^6^ BM cells were plated into dishes with 100 mm diameter in 10 ml of medium and induced by GM-CSF (40 ng/ml) and IL-6 (40 ng/ml) cytokines (R&D Systems, Inc., MN, USA).^42^ The cells were maintained at 37 °C in 5% CO_2_-humidified atmosphere for 4 days in the culture medium with RPMI 1640 (Biosera, Boussens, France), 150 U/ml streptomycin, 200 U/ml penicillin and 10% heat-inactivated FBS (Biosera). After 4-day induction, MDSCs were sorted on a FACS Aria II (Becton Dickinson, San Diego, CA, USA) by using mAb CD11b (clone: M1/70), Ly-6G (clone: RB6-8C5) and Ly-6C (clone: HK1.4) (eBioscience, San Jose, CA, USA). Flow cytometry verified that all of the isolated MDSCs yielded a pure population of more than 90%. The sorted MDSCs were cultured in the same medium as described above.

### Treatment

For *in vivo* study, the mice were administrated with 0.5 ml 10% rapamycin suspension (1 ml Sirolimus oral solution (50 mg sirolimus:50 ml solution, Huadong Medicine, Hangzhou, China) suspended in 9 ml saline) by gastric lavage at 24 h, 1 h pre-operation and 12 h post-operation. *In vitro*, MDSCs were treated by 100 nM rapamycin (Sigma-Aldrich (Shanghai, China) Trading Co., Ltd) 4 h before adoptive transfer, mixed lymphocyte reaction or flow cytometry were performed. When used for adoptive transfer, 2 × 10^6^ MDSCs were washed twice, resuspended in 200 *μ*l PBS and injected in mice via tail vein using an insulin syringe.

### Renal function assay

The whole blood was drawn from the heart by using 1 ml syringe and centrifuged at 4 °C, 3000 r.p.m., for 20 min to obtain serum samples. The level of serum creatinine and blood urine nitrogen were measured by using QuantiChrom Creatinine Assay Kit and QuantiChrom Urea Assay Kit (BioAssay Systems, Hayward, CA, USA) according to the manufacturer's instructions.

### Histopathology

The H&E staining was performed to assess the severity of kidney injury. The tissue sections (200-fold magnification) were blind-labeled and reviewed by two renal pathologists. Renal tissue damage was graded by the percentage of tubular injury, and the following histological scoring system was used to estimate the damage: 0 (<1%), 1 (1% to 10%), 2 (11% to 20%), 3 (21% to 40%), 4 (41% to 60%), 5 (61% to 75%) and 6 (>75%). The scores represent the severity of tubular injury (including proximal tubule brush border loss, formation of casts, cell swelling or vacuolization and cell necrosis). Scores in the range from 1 to 2 represent mild injury, whereas scores in the ranges from 3 to 4 and 5 to 6 represent moderate and severe injuries, respectively, as we described before.^43^

### Immunohistochemistry analysis

Immunohistochemical staining of CD4 and CD8 was performed on paraffin sections using a DAKO ChemMate EnVision Detection Kit (DAKO, Glostrup, Denmark). Antigen retrieval was performed using 10 mM sodium citrate buffer (pH 6.0) in a steam bath maintained by high-power microwave for 20 min. Antigen was blocked by peroxidase-blocking reagent. The sections were labeled with primary antibodies at 4 °C overnight or by a normal rabbit immunoglobulin G (DAKO) at the same concentration of primary antibody as the negative control. The dilution ratio for each antibody was shown as follows: 1:500 for CD4 (clone: EPR19514, Abcam, Cambridge, MA, USA), 1:200 for CD8*α* (clone: YTS 169AG 101HL, Abcam). The antibody binding was revealed by using 3,3-diaminobenzidine (DAB). The prepared slides were photographed using an ECLIPSE E600 microscope with an attached Digital Sight camera (Nikon, Tokyo, Japan). The number of positive cells in the digital images (200-fold magnification) was counted using the ImageJ software. The positive cells were counted in at least 10 different fields in each slide and the numbers of positive cells were averaged.

### ISEL apoptotic cells detection

ISEL apoptotic cells were detected by using a TUNEL (TdT-mediated dUTP Nick-End Labeling) Label Mix (Roche, Mannheim, Germany). Paraffin-embedded tissue sections of 4*μ*m were digested by 40*μ*g/ml of proteinase K (Roche) at 37 °C for 30 min. The addition of 50 *μ*l TUNEL reaction mixture (5 *μ*l TUNEL-Enzyme solution and 45 *μ*l TUNEL-Label solution) on the sample was followed by the addition of lid and incubation for 60 min at 37 °C in a dark, humidified chamber. To be converted into a colorimetric signal, the sample was added with 50*μ*l TUNEL AP and then incubated in a humidified chamber for 30 min. After the addition of 50–100 *μ*l substrate solution, the sample was incubated for another 10min at 15 to 25 °C. The samples were then mounted under glass coverslips and analyzed under light microscope at 200-fold magnification. The rate of apoptotic cells in the kidney was defined as the ratio of apoptotic cells to total cells at 20 non-overlapping visual fields.

### Flow cytometry

The single cell suspension of spleen and kidney was prepared as previously described.^44^ Naive T cells were isolated from mononuclear splenocytes by magnetic-activated cell sorting according to the manufacturer's instructions (Miltenyi Biotec, Auburn, CA, USA) and activated by the stimulation with anti-CD3 antibody (5 *μ*g/ml) and soluble anti-CD28 antibody (1 *μ*g/ml; eBioscience, San Diego, CA, USA) for 3 days. Thereafter all single cells were resuspended in staining buffer (BD Bioscience, San Diego, CA, USA). The following monoclonal antibodies were added in the single-cell suspension according to manufacturers' protocols: CD11b (clone: M1/70, eBioscience, San Jose, CA, USA), Ly-6G (clone: RB6-8C5, eBioscience, San Jose, CA, USA), Ly-6C (clone: HK1.4, eBioscience, San Jose, CA, USA),CXCR2 (clone: 242216, R&D Systems), CD3e (clone: 145-2C11, BD Bioscience), CD4 (clone: RM4-5, BD Bioscience), CD8 (clone: 53-6.7, BD Bioscience), IFN-*γ* (clone: XMG1.2, BD Bioscience), and IL-17a (clone: N49-653, BD Bioscience). The samples were incubated at 4 °C for 1 h in a photophobic condition and then washed twice with staining buffer before they were tested on a FACS Aria II (Becton Dickinson).

For Th1 or Th17 differentiation, CD4^+^ T cells were sorted and then activated with anti-CD3 and anti-CD28 monoclonal antibodies plus IL-4 (5 *μ*g/ml)+IL-2 (2 ng/ml) or IL-4 (5 *μ*g/ml)+IFN-*γ* (5 *μ*g/ml)+TGF-*β* (1 ng/ml)+IL-6 (20 ng/ml) for 3 days. For CD8^+^ T cells differentiation, CD8^+^ T cells were sorted and then activated with anti-CD3 and anti-CD28 monoclonal antibodies plus IL-2 (100 U) for 3 days. Then Th1, Th17 or CD8^+^ T cells were stimulated with PMA (50 ng/ml)+Ionomycin (500 ng/ml)+Golgistop for 4 h. Then CD4, CD8, IFN-*γ* and IL-17a FACS antibodies were stained for flow cytometry.

### RNA isolation and real-time RT-qPCR

Total RNA isolation was performed using TRIzol reagent according to the manufacturer's instructions (Invitrogen, Waltham, MA, USA). The quality and integrity of RNA were evaluated via A260/A280 ratio and 18 s/28 s band by agarose electrophoresis. Then total RNA was reversed to first-strand cDNA using RevertAid First Strand cDNA Synthesis Kit (Thermo Fisher Scientific, Inc., Waltham, MA, USA). RT-qPCR was performed in duplicate using All-in-One qPCR Mix (GeneCopoeia, Inc., Rockville, MD, USA). An Eppendorf Mastercycler Realplex PCR system was used for quantitative PCR under the following conditions: initial denaturation was performed at 95 °C for 10 min, followed by 40 cycles of denaturation at 95 °C for 10 s, annealing at 60 °C for 20 s and extension at 72 °C for 15 s. GAPDH was used as an internal control to normalize differences in the amount of total RNA in each sample. The threshold cycle (Ct) values were analyzed using the comparative Ct (−ΔCt) method. The expression level of target genes was obtained by normalizing to the endogenous reference and relative to control. Primer sequences are listed in Supplementary Table S1.

### Western blotting

The −80 °C stored kidney tissue samples were mixed with RIPA (9:1) and then homogenized with the aid of a mortar and pestle. Tissue lysate was then transferred to sterile microcentrifuge tubes and centrifuged at 12 000 g at 4 °C for 15 min. The prepared protein samples were heated at 100 °C for 5 min. The proteins were separated on SDS-polyacrylamide gels and transferred to polyvinylidene difluoride membranes. The membranes were blocked with 5% (w/v) milk for 1 h and incubated overnight at 4 °C with *β*-actin (1:1000, Cell Signaling Technology, Danvers, MA, USA), Argniase-1 (1:1000, Cell Signaling Technology), iNOS (1:1000, Cell Signaling Technology) and Runx1 (1:500, Cell Signaling Technology). The membranes were then incubated with goat-derived anti-rabbit IgG (1:10 000) for 1 h and detected with enhanced chemiluminescence reagents (Thermo Fisher Scientific). The bands were visualized and detected by using Alpha Imager (Alpha Innotech, San Leandro, CA, USA).

### Serum cytokine assay

To measure the level of cytokines in the serum, mouse blood sample (*n*=5/group) was collected into tubes, followed by centrifugation for 10 min at 3000 g. The supernatant was collected and stored at −80 °C until further analysis. Serum levels of IL-1*β*, IL-6, IFN-*γ* were measured using a Cytokine Mouse 20-Plex Panel for Luminex Platform (LMC0006, Invitrogen) and serum level of TGF-*β*1 was measured using Mouse TGF-*β*1 ELISA Kit (ab119557, Abcam) in accordance with the manufacturer's protocol, respectively.

### Immunosuppressive function assay *in vitro*

Mononuclear cells were obtained from spleens of normal C57BL/6 mice by Ficoll density gradient centrifugation. CD4^+^ T and CD8^+^ T cells were isolated from mononuclear cells by magnetic-activated cell sorting according to the manufacturer's instructions (Miltenyi Biotec, Auburn, CA, USA). The sorted CD4^+^ T cells were plated at a density of 2 × 10^5^ cells/well in 96-well plates with RPMI 1640 medium and stained with CFSE by using CellTrace CFSE Cell Proliferation Kit (Invitrogen) for 30 min. Then the stained T cells were stimulated with anti-CD3 antibody (5 *μ*g/ml) and soluble anti-CD28 antibody (1 *μ*g/ml) for 3 days (eBioscience, San Diego, CA, USA). The activated T cells were co-cultured with BM-derived MDSCs with or without rapamycin treatment at the ratio of 1:10 and 1:3 for 5 days before their proliferation assay was performed by flow cytometry using FACS Aria II (Becton Dickinson). The mean fluorescence intensity (MFI) of CFSE dye is negatively correlated with the proliferative activity of T cells. So the increase of MFI could quantify the suppressive effect on T-cell proliferation.

### Statistical analysis

The data were analyzed using GraphPad Prism 5 software or Statistical Program for Social Sciences software 18.0 (SPSS Inc., Armonk, NY, USA). Quantitative variables were analyzed by one-way ANOVA (among three or more groups), two-tailed independent *t*-test (between two groups) and expressed as means±S.D. *P*<0.05 was considered statistically significant.
