# Supplementary material for: The mTOR signal regulates myeloid-derived suppressor cells differentiation and immunosuppressive function in acute kidney injury
Source: Cell Death Dis. 2017 Mar 23;8(3):e2695–. doi: 10.1038/cddis.2017.86 (PMC5386577; doi:10.1038/cddis.2017.86)
Supplement: Supplementary Table S1 [file cddis201786x1.docx]

**Table S1. Primer Sequences Used for RT-qPCR**

| Genes | Forward primer | Reverse primer |
| --- | --- | --- |
| GAPDH | 5’-GACTTCAACAGCAACTCCCAC-3’ | 5’-TCCACCACCCTGTTGCTGTA-3’ |
| CXCL1 | 5’-ACTGCACCCAAACCGAAGTC-3’ | 5’-TGGGGACACCTTTTAGCATCTT-3’ |
| CXCL2 | 5’-CCAACCACCAGGCTACAGG-3’ | 5’-GCGTCACACTCAAGCTCTG-3’ |
| CXCL3 | 5’-CTCAGTGCTGCACTGGTCCT-3’ | 5’-CACTGACAGCGCAGCTCACT-3’ |
| CXCL7 | 5’-GCTGCAGATGTACGAATACC-3’ | 5’-CACATCAGCACAGTGAACTC-3’ |
| IL-1β | 5’-CAACCAACAAGTGATATTCTCCATG-3’ | 5’-CTCTCTGAAGGACTCTGGCTTTG-3’ |
| IL-6 | 5’-GAAATGATGGATGCTACCAAACTG-3’ | 5’-CTCTCTGAAGGACTCTGGCTTTG-3’ |
| IFN-γ | 5’-CTTCCTCATGGCTGTTTCTGG-3’ | 5’-ACGCTTATGTTGTTGCTGATGG-3’ |
| TGF-β1 | 5’-CCGCAACAACGCCATCTATG-3’ | 5’-CTCTGCACGGGACAGCAAT-3’ |
| Foxp3 | 5’-GGCCCTTCTCCAGGACAGA-3’ | 5’-GGCATGGGCATCCACAGT-3’ |
| iNOS | 5’-CACCAAGCTGAACTTGAGCG-3’ | 5’-CGTGGCTTTGGGCTCCTC-3’ |
| Arg-1 | 5’-CCAGAAGAATGGAAGAGTCAGTGT-3’ | 5’-GCAGATATGCAGGGAGTCACC-3’ |
| Runx1 | 5’-CGCCACAAGTTGCCACCTAC-3’ | 5’-GGTGCGGGCTGACCCTCAT-3’ |
